# An Indoor Localization Method for Pedestrians Base on Combined UWB/PDR/Floor Map

**DOI:** 10.3390/s19112578

**Published:** 2019-06-06

**Authors:** Fei Liu, Jian Wang, Jixian Zhang, Houzeng Han

**Affiliations:** 1 School of Environment Science and Spatial Informatics, China University of Mining and Technology (CUMT), Xuzhou 221116, China; pntrc@cumt.edu.cn; 2 School of Geomatics and Urban Spatial Informatics, Beijing University of Civil Engineering and Architecture (BUCEA), Beijing 102616, China; hanhouzeng@bucea.edu.cn; 3 National Quality Inspection and Testing Center for Surveying and Mapping Products, Beijing 100830, China; zhangjx@casm.ac.cn

**Keywords:** UWB, PDR, Floor Map, EKF, Pedestrians Indoor Localization

## Abstract

This paper propose a scheme for indoor pedestrian location, based on UWB (Ultra Wideband)/PDR (Pedestrian Dead Reckoning) and Floor Map data. Firstly, a robust algorithm that uses Tukey weight factor and a pathological parameter for UWB positioning is proposed. The ill-conditioned position problem is solved for a scene where UWB anchors are placed on the same elevation of a narrow corridor. Secondly, a heading angle-computed strategy of PDR is put forward. According to the UWB positioning results, the location of pedestrians is mapped to the Floor Map, and 16 possible azimuth directions with 22.5° interval in this position are designed virtually. Compared to the heading angle of PDR, the center direction of the nearest interval is adopted as the heading. However, if the difference between the head angles of PDR and the nearest map direction is less than five degrees, the heading angle of PDR is regarded as the moving heading. Thirdly, an EKF (Extended Kalman Filter) algorithm is suggested for UWB/PDR/Floor Map fusion. By utilizing the positioning results of UWB, PDR, and the possible heading angle of Floor Map, high precision positioning results are acquired. Finally, two experimental scenarios are designed in a narrow corridor and computer room at a university. The accuracy of pedestrian positioning when all the data are available is verified in the first scenario; the positioning accuracy of a situation where part of UWB is unlock is verified in the second scenario. The results show that the proposed scheme can reliably achieve decimeter-level positioning.

## 1. Introduction

People live and work indoors more than 90% of their lives [1]. The provision of accurate indoor positioning services is a real demand for the security of large buildings, such as the interior of the building, airport halls, exhibition halls, warehouses, supermarkets, libraries, underground parking lots, mines, and other environments, especially in disaster sites, high-rise building rescue and other industries. The research on related theories and implementation schemes of indoor pedestrian location has attracted extensive attention and has become a research hotspot in recent years. At present, the common devices used for indoor positioning technologies include infrared [2,3], Ultrasonic [4,5], WLAN (Wireless Local Area Networks) [6,7], Bluetooth [8,9], UWB [10,11], LED (Light Emitting Diode) [12,13], Geomagnetic sensors [14,15], and so on [16,17]. The interior is affected by the layout of the building, the internal structure, materials, decoration, temperature, humidity, the distribution of the flow of people, the pattern of the indoor environment, the placement of goods, frequent changes in the dynamics of the flow of people, and so on. It makes the problem of indoor target location very challenging. Although a single technology has a certain availability, it is basically in the completion of a simple positioning task, and there is still a certain gap for the real realization of the goal of an indoor “intelligent space”.

UWB technology has low power consumption, good anti-multipath effect, high security, low system complexity, high positioning accuracy, and so on [18]. PDR positioning algorithm is an autonomous positioning method. Its primary principle is to obtain the motion direction and step information of firefighters in real time by using an accelerometer, gyroscope and geomagnetic meter, so as to calculate the position information of the moving target [19]. PDR positioning has high positioning accuracy in a short time, but it can only obtain relative positioning results, and there are cumulative errors. Therefore, the pedestrian location method based on UWB/PDR combination stands out among many indoor positioning technologies. Chen et al. [20] used an unscented Kalman filter to fuse inertial sensors and UWB data. The experimental results show that the positioning error of this method is reduced by 74.5% and 43.5% compared to that of PDR and UWB respectively. This method is suitable for a situation where UWB base stations are evenly distributed around pedestrians, but in a narrow corridor; UWB positioning will have singular solutions, which will lead to the reduction of positioning accuracy. Tian et al. [21] proposed using only one UWB anchor node at an unknown location. The proposed PTS has limitations, namely that the anchor position is estimated only once at the initial stage of tracking. The subsequent fusion-tracking algorithm is affected by the accuracy in anchor location estimation. Giarré, L. et al. [22] proposed a localization system for human beings, by exploiting both PDR and UWB data, which allows to correct the heading, so improving the overall system performance. However, heading correction depends on the number and distribution of UWB. Wang, J. et al. [23] proposed a scheme for indoor positioning by fusing the floor map, WiFi and smartphone sensor data. This method can solve the problem of INS (Inertial Navigation System) heading error accumulation by using map constraints, but the accuracy of WiFi positioning datum is low. For instance, the experiment results of Bachtler, M. et al. [24] show that this approach can achieve an average error of 3.2 m and 90% accuracy of 4.1 m. Li, X. et al. [25] proposed a UWB/PDR fusion algorithm which is based on the extended Kalman filter. Experimental results show that the algorithm is robust to the intermittent noise, continuous noise, signal interruption, and other abnormalities of the UWB data. However, if fewer UWB anchors are used, the PDR heading angle error will be difficult to control. Hartmann, F. et al. [26] put forward a hybrid localization concept and experimental results by the combination of an INS and a spatial non-uniform UWB-network. However, each location network needs eight UWB anchors, which is a large number; in addition, in the area without anchors, the error accumulation of INS equipment is difficult to control. The state-of-the-art research results of Ref. [27] show that the magnetic positioning system (MPS) can lead to a mean positioning error of the order of 0.6 m in a static scene, which is comparable with the commercial UWB system. However, this level of positioning accuracy of the system requires evenly-distributed anchor equipment. Zampella, F. et al. [28] put forward a constraint approach for UWB and PDR fusion that is able to estimate the position of a person with a limited error growth for the dead reckoning system and a better position estimate between position updates for the UWB system. In addition, the RMSE of the constraint UWB/INS is about 0.2–0.5 m. Gentner, C. et al. [29] presented a novel iterative pedestrian localization algorithm using an UWB positioning system without the necessity to have prior information on the UWB anchor positions, and the accuracy of the pedestrian location in the order of 20 cm. Corrales, J.A. et al. [30] developed a hybrid tracking system of human operators using IMU/UWB data fusion by a kalman filter. The obtained animation shows that limb movements are very accurate and the global localization of the human operator is appropriate. Benini, A. et al. [31] introduced an approach for the indoor localization of a mobile agent based on Ultra-WideBand technology and a low-cost IMU (Inertial Measurement Unit) using a biased EKF as a possible technique to improve the localization. In a room which has an area of about 9.5 m × 7.5 m, there are four UWB anchors deployed near the corners. The obtained results show that the error amplitude is less than 0.15 m. Gonza’lez et al. [25,32] used a particle filter (PF) algorithm to fuse the data of UWB, IMU, and odometer, achieving good positioning stability under the NLOS conditions. However, compared to EKF, PF can achieve optimal estimation accuracy in high dimensional and computational complexity problems. For low dimensional problems, EKF can achieve roughly the same estimation accuracy compared to a PF [33,34]. Foxlin [35] applies zero-velocity updates (ZUPTs) as pseudomeasurements into the EKF, which correct the velocity error after each stride, breaking the cubic-in-time error growth and replacing it with an error accumulation that is linear in the number of steps. However, yaw (heading) and the yaw gyro bias are not observable from zero-velocity measurements.

**Figure 1:**
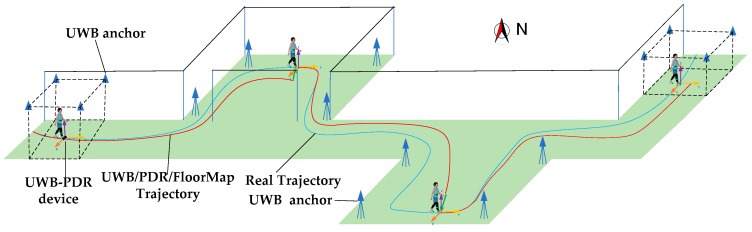
System concept of the proposed pedestrian navigation system based on UWB/PDR/Floor Map.

In summary, at present, there are usually several problems in using UWB and PDR fusion methods: (1) more UWB anchors are needed; (2) in narrow corridors, the results of UWB positioning are morbid; (3) with the increase of time, the heading error calculated by INS sensor accumulates. In order to solve the above problems, this paper proposes a method based on a combined EKF Algorithm for UWB/INS/Floor Map. The application scenario is shown in Figure 1.

Figure 1 is a system concept and novelty of this paper. Firstly, a sparse UWB network is constructed, and UWB anchors are placed at the end of narrow corridors and rooms. The problems of pedestrian motion initialization and positioning error correction are solved. The signals of UWB anchors do not need to cover all areas. Secondly, a pedestrian who wears an UWB-PDR device walks indoor where the floor maps are known. In the process of pedestrian positioning, UWB can be used as an absolute space reference, PDR equipment can be used to encrypt positioning data and blind UWB regional dead reckoning, and the azimuth and spatial coordinates of indoor map can be used to modify the heading angle and position obtained by PDR.

In our research, the method for pedestrian motion initialization and positioning error correction are studied with a sparse UWB network in narrow corridors and indoors, and the ill-conditioned position problem of UWB is solved for a scene where UWB anchors are placed at the same elevation of a narrow corridor. A heading angle computed strategy of PDR is put forward based on the UWB positioning results and Floor Map. The PDR method is used to solve the problem of continuous positioning in the environment where the UWB signal is weak or not. Then, an EKF fusion algorithm for three kinds of data is used to realize the high-precision positioning of pedestrians in a large range of complex indoor environments. The experimental results show that in a narrow and long indoor environment with a large area, the positioning accuracy can be maintained at the order of 0.4 m with the UWB signal unlocked for a long time. This is in a similar order of magnitude compared to the positioning results of the above literature of which UWB signal is locked in real time. So this scheme can not only reduce the number of UWB anchors and reduce the cost and the preparation of UWB anchors deployed, but also ensure high positioning accuracy.

This paper is organized as follows. In Section 2, the theory of localization algorithm is first presented, including an improved robust UWB positioning algorithm, a PDR positioning algorithm and strategy, and a combined fusion algorithm for a UWB/PDR/Floormap. Subsequently, two experiments of UWB in full use and part unlocking are carried out; the positioning results are presented and discussed, and the innovations of the paper are summarized.

## 2. Localization Algorithm

**Figure 2:**
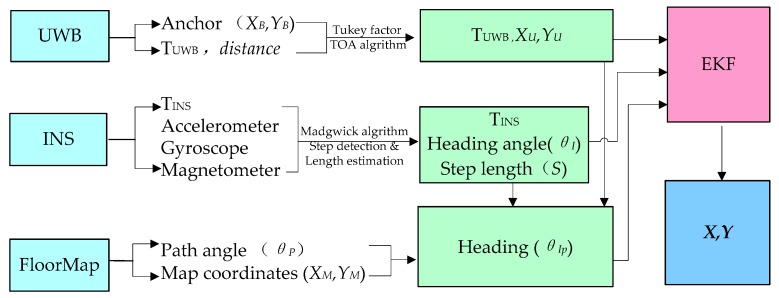
The overall flowchart.

Figure 2 shows the data fusion of the proposed hybrid pedestrian localization and navigation system. Firstly, the robust least square method and ill-conditioned equation solution are used to obtain the accurate UWB positioning results. Secondly, the 9-axis INS sensor is used to calculate the pedestrian heading information based on a madgwick algorithm; meanwhile, the heading angle will be modified by the information of the floor map. The step length algorithm is used to estimate the step size. Finally, an EKF model is used to fuse UWB, PDR and Floor Map data to achieve the position of pedestrian indoor motion.

### 2.1. An UWB Localization Algorithm

UWB is a carrier-free communication technology, which has the characteristics of strong penetration, low power consumption, good anti-multipath effect, high security, low system complexity, and high positioning accuracy. We can use its sub-nanosecond ultra-narrow pulse to perform accurate indoor positioning.

TOA [27] (Time of Arrival) positioning technology is a location method of UWB. By measuring the time of the signal from the tag to three or more UWB anchors, the distance from the tag to the anchors can be obtained. Draw three or more circles with the anchor point as the center and the distance as the radius respectively. The intersection of the circles is the position of the tag. The Equation is as follows:

(1) $$t_{i}=\tau_{i}+t_{0}=d_{i}/c+t_{0}=\sqrt{{\left(x_{i}-x\right)}^{2}+{\left(y_{i}-y\right)}^{2}+{\left(z_{i}-z\right)}^{2}}/c+t_{0}$$

In Equation (1), $t_{0}$ is the time when the signal is transmitted from a tag. $t_{i}$ is the receiving time of the signal by an anchor. $\tau_{i}$ is the propagation time of the signal from the tag to an anchor. $d_{i}$ is the distance from the tag to an anchor. $\left(x_{i},y_{i},z_{i}\right)\;\mathrm{and}\;\left(x,y,z\right)$ are the coordinates of the anchor and the tag, respectively. In the three-dimensional coordinate solution, Equation (1) can be transformed into the form of Equation (2):

(2) $$d_{i}=\sqrt{{\left(x_{i}-x\right)}^{2}+{\left(y_{i}-y\right)}^{2}+{\left(z_{i}-z\right)}^{2}}\;\left(i=1,2,\ldots ,n\right)$$

$X={\left(x,y,z\right)}^{T}$ are the coordinates of the tag. Usually, the number of anchors is at least three or more, so there are redundant observations in the calculation of tag coordinates, and the least square adjustment calculation can be carried out. The Equation is as follows:

(3) AX=L

A, L can be calculated from the base station coordinates and the distance from the label to the base station, such as Equations (3) and (4).

(4) $$A=\left[\begin{array}{ccc} x_{\left(i+1\right)}-x_{1,} & y_{\left(i+1\right)}-y_{1,} & z_{\left(i+1\right)}-z_{1} \end{array}\right]\;\left(i=1,2,\ldots ,n\right)$$

(5) $$\{\begin{array}{c} L=0.5\times \left[{\left(x_{i+1}\right)}^{2}-{\left(x_{1}\right)}^{2}+{\left(y_{i+1}\right)}^{2}-{\left(y_{1}\right)}^{2}+{\left(z_{i+1}\right)}^{2}-{\left(z_{1}\right)}^{2}+{\left(d_{1}\right)}^{2}-{\left(d_{i+1}\right)}^{2}\right]\\ \left(i=1,2,\ldots ,n\right) \end{array}$$

V is the residual error of the observation, such as Equation (6)

(6) V=AX−L

(7) $$X={\left(A^{T}PA\right)}^{-1}A^{T}PL$$

The tag coordinates can be calculated by Equation (7). Here, P is the unit weight matrix. In this method, the observation with different residual values cannot be treated differently; it is almost impossible to eliminate observations with large residual errors.

Tukey weight factor [36] estimate method is the maximum likelihood estimation with an obsolete area, whose influence function is bounded, which is not sensitive to small changes in the middle part of the observation values, but is sensitive to the great changes. According to the magnitude of the residual error V, the weight factor P will be adjusted adaptively. The Tukey weight factor function is shown in Equation (8).

(8) $$P=\omega \left(u\right)=\{\begin{array}{c} \begin{array}{cc} {\left(1-u^{2}\right)}^{2}, & \left|u\right|\leq 1 \end{array}\\ \begin{array}{cc} 0, & \left|u\right|>1 \end{array} \end{array}$$

(9) $$\{\begin{array}{c} u=\frac{L-A^{T}\hat{X}}{c\cdot MAD}\\ MAD=med\left|L-A^{T}\times {\hat{X}}_{tukey}\right| \end{array}$$

Because UWB anchors are deployed on the same horizontal plane, the coefficient matrix of the normal equation is close to ill-conditioned. In order to improve this situation, the regularization factor λ is introduced to solve the problem. As shown in Equation (10).

(10) $$X={\left(A^{T}PA+\lambda \right)}^{-1}A^{T}PL$$

### 2.2. A PDR Localization Algorithm

PDR is a method to calculate the walking track by counting and measuring the number, step length and direction of the pedestrian, which are the key factors for positioning.

#### 2.2.1. Heading Angle Calculated Algorithm and Strategy

In order to collect the heading data of pedestrians, the acceleration, angular velocity and magnetic field intensity data of pedestrian motion are obtained using a 9-axis INS sensor. The Madgwick algorithm [37] is used to fuse the sensor data, and the information for the roll, pitch and heading of the pedestrian in the walking process is calculated, as shown in Equation (11).

(11) {qESest,t=a1qESω,t+a2qES∇,tα1+α2=1,0≤α1≤1,0≤α2≤1

where qESω,t is the attitude calculated by the gyroscope at time *t*, and qES∇,t is the attitude calculated by the accelerometer and the magnetometer at time *t*. $\alpha_{1}$ and $\alpha_{2}$ are weighting coefficients.

As we all know, the error of positioning results calculated with INS data will gradually increase over time. In addition, the heading error is large, which is difficult to meet the needs of PDR positioning. In this paper, the 16 wind direction map is introduced, and a heading angle calculation strategy is worked out.

**Figure 3:**
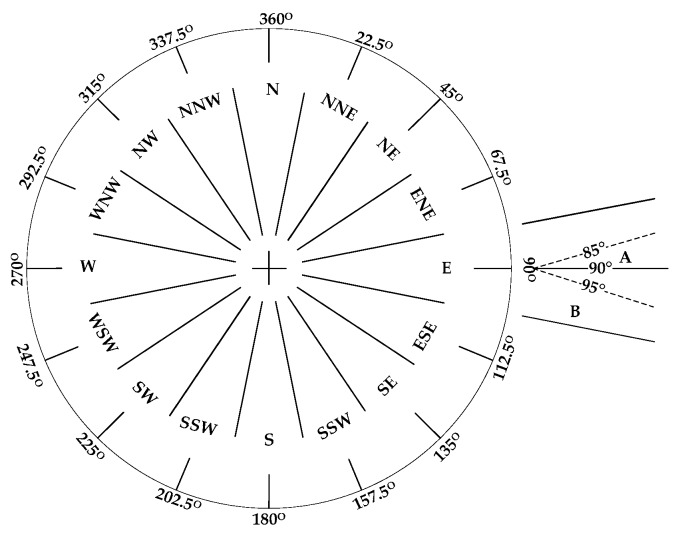
Sixteen wind direction map.

Figure 3 is a sixteen wind direction map that is introduced to paper as a virtual map heading. There are 16 equal direction intervals. Each interval is 22.5 degrees. The center direction of each interval is adopt as the moving heading of a pedestrian. That is to say, when a person walks indoors, there are 16 possible directions.

**Figure 4:**
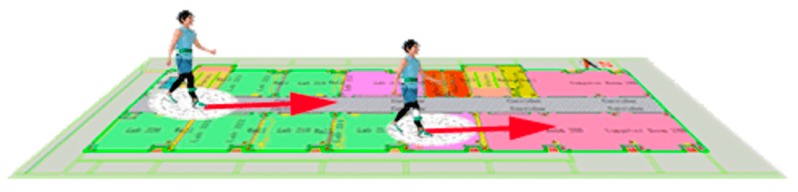
Heading angle calculated strategy with floor map.

From Figure 4, according to the UWB positioning results, the location of a pedestrian is mapped to the Floor Map, and 16 possible directions of equal intervals in this position are designed virtually. The starting direction is the North direction of the map. Compared to the heading angle of INS, the center direction of the nearest interval is adopt as the heading. However, if the difference between the heading angle of INS and the nearest map direction is less than five degrees, the heading angle of INS is adopted as the moving heading. For example, A and B present two moving heading angles in Figure 3. A is between 85 and 90 degree. Therefore, A is adopted as the moving heading. B is more than 95 degrees, and 90 degrees is adopted as the moving heading. With this method, we can limit the error of the heading angle gradually to increase.

#### 2.2.2. Step Length Calculated Algorithm

Practically, step length varies from one person to another [38]. There are acceleration fluctuations in the front and back, up and down; from foot lifting to landing, the center of gravity rises to fall, and the vertical acceleration data show the wave peak and trough curve. Gait detection can be realized by analyzing the change of acceleration. The average success rate of gait recognition is 99.2% when walking at normal speed, and 96.7% when running at different speeds [39]. To detect a user step, we use a synthetic acceleration at time t,a(t), calculated from the root mean square sum of all three acceleration values as

(12) $$a\left(t\right)=\sqrt{a_{x}^{2}\left(t\right)+a_{y}^{2}\left(t\right)+a_{z}^{2}\left(t\right)}-g$$

where the constant component g represents the earth’s gravity acceleration (9.8 m/s^2^).

Here, we use the step length calculated model which was proposed in Ref. [40] and verified in Ref. [41] to estimate the user step length. The *k*th step length, *S*, with the detected step time $t_{k}^{s}$, can be calculated from the acceleration measurements as shown in Equation (13).

(13) $$S=\eta \cdot \sqrt[{4}]{\alpha_{max}\left(t\right)-\alpha_{min}\left(t\right)},\;t_{k-1}^{s}<t\leq \;t_{k}^{s}$$

where $\alpha_{max}\left(t\right)$ and $\alpha_{min}\left(t\right)$ are the maximum and minimum values of acceleration during the time t, respectively. The coefficient η, which can be determined through calibrations, is the ratio of the real and estimated distance of a reference path as

(14) $$\eta =d_{real}/d_{estimated}$$

The current user position, $P_{u,k}$, is then propagated by adding the estimated current step length,$\;S_{k},$ to the previous location points with the estimated heading orientation, $\theta_{k}$, according to

(15) $$P_{u,k}=\left[\begin{array}{c} E_{k}\\ N_{k} \end{array}\right]=\left[\begin{array}{c} E_{k-1}+S_{k}\cdot \sin\left(\theta_{k}\right)\\ N_{k-1}+S_{k}\cdot \cos\left(\theta_{k}\right) \end{array}\right]$$

where $E_{k}$ and $N_{k}$ represent the East and North coordinates, respectively.

### 2.3. A Combined EKF Algorithm

The absolute positioning accuracy of UWB is high and the position information is updated once in 1–2 s, which can be used as the absolute datum of pedestrian positioning. INS can output original observation data more than 100 Hz. PDR positioning algorithm based on INS data has the characteristics of strong autonomy and high precision in a short time, and can be used as a positioning supplement to blind the UWB environment. However, the heading accuracy of PDR calculated by INS decreases gradually with the passage of time. If a person walks in the environment of a known indoor map, the possible indoor pedestrian forward direction can be extracted from the map and can be adopted as heading constraint parameters. For the fusion of all above data, an EKF fusion algorithm [39] based on UWB/PDR/Floor Map is adopted.

Firstly, the acceleration data in inertial measurements are used to judge the static or moving state. At the beginning of positioning, the pedestrian stands still for a few seconds, the gross error of UWB positioning which results in static state is eliminated, the mean value is calculated as the starting position of PDR, and then the PDR calculation is carried out. The Kalman filter is adaptively determined by the model noise considering the motion information. The state-space models are

(16) $$\;X_{k+1}=\varphi \left(k,X_{k}\right)+w_{k}$$

(17) $$Z_{k}=H(k,X_{k})+v_{k}$$

where $w_{k}$ and $v_{k}$ are independent, zero mean, Gaussian noise processes of covariance matrices $Q_{k}$ and $R_{k}$, respectively. The position error dN,dE, the difference of moving distance ds and the course difference dθ are taken as the state variables of the filter system, that is, the state vector of the system is:

(18) X=[dN,dE,ds,dθ]

When the location of the UWB system is updated, the position difference between the UWB and PDR systems is used as the system observation, that is:

(19) $$Z={\left[\Delta N,\Delta E\right]}^{T}={\left[N_{k,u}-N_{k,p},E_{k,u}-E_{k,p}\right]}^{T}$$

In the Equation (19), (ΔN,ΔE) denotes the position difference between the two positioning systems at the time *k*$;\;(N_{k,u},E_{k,u})$ is the positioning result of the UWB system at time *k*; and $\left(N_{k,p},E_{k,p}\right)$ is the position information calculated according to the PDR algorithm at time *k*.

Because of the different update rates between the UWB and PDR systems, system time synchronization is particularly important. In our UWB-PDR tag device, there is a 4G communication module on board, which will be able to calibrate system time to UTC time in real time through access to the internet. So, a unified time benchmark is provided to UWB and PDR systems.

When both systems update at the same time, the system observation is Equation (19). Once the UWB position results is not updated, as shown in Equation (20), the difference between the system prediction coordinates and the PDR observation coordinates is taken as the system observation variable; the others remain unchanged, and the position of the PDR is recursively corrected.

(20) $$Z={\left[\Delta N,\Delta E\right]}^{T}={\left[\begin{array}{cc} N_{p,k+1}^{-}-N_{p,k}^{-}, & E_{p,k+1}^{-}-E_{p,k}^{-} \end{array}\right]}^{T}$$

(21) $$\{\begin{array}{c} N_{p,k}^{+}=N_{p,k}+dN\\ E_{p,k}^{+}=E_{p,k}+dE \end{array}$$

(22) $$\{\begin{array}{c} N_{k+1}^{-}=N_{p,k}^{+}+\left(s_{k}+ds_{k}\right)\cdot \cos\left(\theta_{k}+d\theta_{k}\right)\\ E_{k+1}^{-}=E_{p,k}^{+}+\left(s_{k}+ds_{k}\right)\cdot \sin\left(\theta_{k}+d\theta_{k}\right) \end{array}$$

Equation (21) uses the position error obtained by filtering to update the pedestrian position at the current time, which is also the final position calculated by the fusion model, and uses the filtering error value to predict the position of the next time (Equation (22)).

Although $\varphi \left(k,X_{k}\right)$ is a nonlinear matrix, this problem can be solved effectively by the expansion of the first-order Taylor Series. Definitions:

(23) $$\varphi_{k+1,k}={\frac{\partial \varphi \left(k,X\right)}{\partial X}|}_{X=X_{k}}$$

(24) $$H_{k}={\frac{\partial H\left(k,X\right)}{\partial X}|}_{X=X_{k}^{-}}$$

So, the state transition matrix is

(25) $$\varphi_{k}=\left[\begin{array}{cc} \begin{array}{cc} 1 & 0\\ 0 & 1 \end{array} & \begin{array}{cc} cos\theta_{k} & -s_{k}\cdot sin\theta_{k}\\ sin\theta_{k} & \;s_{k}\cdot cos\theta_{k} \end{array}\\ \begin{array}{cc} 0 & 0\\ 0 & 0 \end{array} & \begin{array}{cc} 1\; & 0\;\\ 0\; & 1\; \end{array} \end{array}\right]$$

The observation matrix is

(26) $$\;H_{k}=\left[\begin{array}{cc} \begin{array}{cc} 1 & 0 \end{array} & \begin{array}{cc} 0 & 0 \end{array}\\ \begin{array}{cc} 0 & 1 \end{array} & \begin{array}{cc} 0 & 0 \end{array} \end{array}\right]$$

State estimate propagation is

(27) $$\;{\hat{X}}_{k}^{-}=\varphi (k,{\hat{X}}_{k-1}^{-})$$

Error covariance propagation is

(28) $$P_{k}^{-}=\varphi_{k,k-1}P_{k-1}\varphi_{k,k-1}^{T}+Q_{k-1}$$

By analyzing a large number of positioning results data, the error characteristics of the UWB and PDR system are counted, and the empirical values are obtained. The dynamic noise matrix is

(29) $${\;Q}_{k}=\left[\begin{array}{c} \delta_{N}^{2}\;0\;0\;0\\ 0\;\delta_{E}^{2}\;0\;0\\ 0\;0\;\delta_{s}^{2}\;0\\ 0\;0\;0\;\delta_{\theta }^{2} \end{array}\right]$$

Among them, the dynamic noise of the position coordinate and displacement satisfies the Gaussian distribution, that is, $w_{N}\sim N\left(0,\delta_{N}^{2}\right)$,${\;w}_{E}\sim N\left(0,\delta_{E}^{2}\right)$,${\;w}_{s}\sim N\left(0,\delta_{s}^{2}\right)$. The value ${\;w}_{\theta }$ of the heading is larger in a turning walk, and smaller in a straight-line walk. Through the discrimination of pedestrian motion attributes (straight or turn), the dynamic noise value of the variables related to the heading is determined adaptively. Based on a large number of positioning results, the statistical characteristics of positioning errors of UWB and PDR are compared and analyzed, take $\delta_{N}^{2}=\delta_{E}^{2}=1$, $\delta_{s}^{2}=1$. When the pedestrian goes straight ahead, take $\delta_{\theta }^{2}={\left(2^{{}^{\circ}}\right)}^{2}$; when the pedestrian turns forward, take $\delta_{\theta }^{2}={\left(15^{{}^{\circ}}\right)}^{2}$. The measurement noise matrix is

(30) $$R=\left[\begin{array}{cc} R_{w} & 0\\ 0 & R_{w} \end{array}\right],R_{w}=10^{2}$$

Kalman gain matrix is

(31) $$\;G_{k}=P_{k}^{-}H_{k}^{T}{\left[H_{k}P_{k}^{-}H_{k}^{T}+R_{k}\right]}^{-1}$$

State estimate update

(32) $${\hat{X}}_{k}={\hat{X}}_{k}^{-}+G_{k}Z_{k}-H\left(k,{\hat{X}}_{k}^{-}\right)$$

Error covariance update

(33) $$\;P_{k}=\left(I-G_{k}H_{k}\right)P_{k}^{-}$$

Because the start point of UWB is thw same as PDR, so the initial estimate of the state ${\hat{X}}_{0}$ is set to [0 0 0 0]. $P_{0}$ is an empirical value.

In this paper, a fusion method based on displacement is proposed for PDR and UWB positioning results. The basic idea is as follows: Firstly, the distance threshold is set to δS. Secondly, the displacement $S_{PDR}$ during the two nearest UWB sampling times is calculated with the accumulation of steps in this time period, in the process of dead reckoning with inertial sensor data. In addition, the displacement $S_{UWB}$ between the two nearest times can be calculated with the Equation of distance between two points. Thirdly, the adaptive weight is acquired based on the relationship between the absolute difference $\left|S_{UWB}-S_{PDR}\right|$ and the distance threshold δS. When satisfying $\left|S_{UWB}-S_{PDR}\right|<\delta S$, the UWB positioning should be taken as the weighted party, that is, Equation (34) would be used to estimate the current position of the people; otherwise, Equation (35) would be used as the current position feedback algorithm.

(34) $$\{\begin{array}{l} N_{k}=\frac{max\left(w_{1},w_{2}\right)N_{k,u}+min\left(w_{1},w_{2}\right)(N_{k,p}-dN)}{w_{1}+w_{2}}\\ E_{k}=\frac{max\left(w_{1},w_{2}\right)E_{k,u}+min\left(w_{1},w_{2}\right)(E_{k,p}-dE)}{w_{1}+w_{2}} \end{array}$$

(35) $$\{\begin{array}{l} N_{k}=\frac{min\left(w_{1},w_{2}\right)N_{k,u}+max\left(w_{1},w_{2}\right)(N_{k,p}-dN)}{w_{1}+w_{2}}\\ E_{k}=\frac{min\left(w_{1},w_{2}\right)E_{k,u}+max\left(w_{1},w_{2}\right)(E_{k,p}-dE)}{w_{1}+w_{2}} \end{array}$$

Among them, at time $k,\;\left(N_{k},E_{k}\right)$ represents the output coordinates of the fusion system.$\;\left(N_{k,u},E_{k,u}\right)$ is the output coordinates of the UWB system. $\left(N_{k,p},E_{k,p}\right)$ is the output coordinates of the PDR system. $\left(w_{1},w_{2}\right)$ expresses the weight. The positioning result is the best when $w_{1}=0.3$ and $w_{2}=0.7$ respectively, measured by the experimental data.

## 3. Experimental and Summary

### 3.1. Experimental Verification

#### 3.1.1. Introduction of Experimental Environment

**Figure 5:**
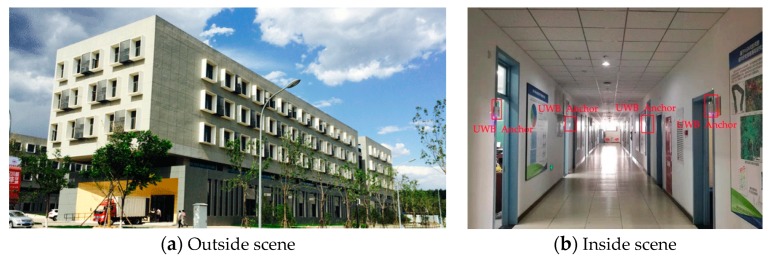
Pedestrian indoor positioning experiment scene.

The experiment was carried out in the School of Surveying and Mapping and Urban Spatial Information of Beijing University of Civil and Architectural. A narrow corridor on the second floor and a laboratory are used in this experiment. The length of the corridor is about 65 m, and the width is about 3 m. The area of the laboratory is about 6.2 m × 8.2 m. The outside and inside scenes are shown in Figure 5.

**Figure 6:**
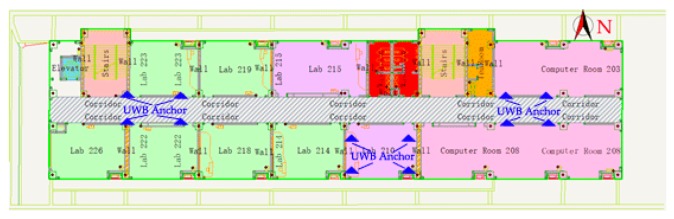
Floor Map of the experiment environment.

There are two groups of UWB anchors that were placed on the wall of the corridor, and another one-group anchor was placed on the floor surface of the laboratory, as shown in Figure 5b and Figure 6. Figure 6 is the indoor map of the experiment scene.

**Figure 7:**
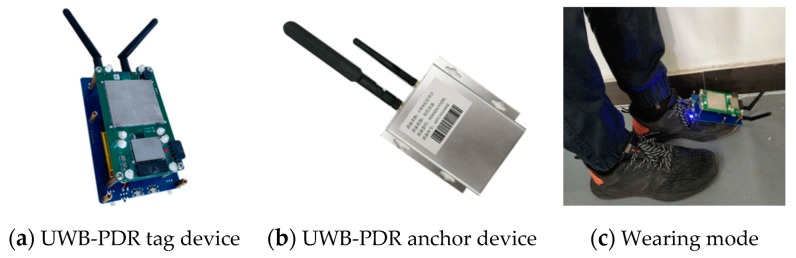
UWB/PDR Positioning equipment and wearing mode.

In the research process of this paper, an INS sensor is developed by using an MEMS 9 axis accelerometer, gyroscope and magnetometer of MPU9250 from InvenSense Company (Sunnyvale, CA, USA). The UWB positioning module is developed by using a UM100 chip (Shanghai uPosition information technology Co., Ltd., Shanghai, China). with ranging accuracy of about 10 cm, and the positioning accuracy can reach 15~30 cm. The device and wearing mode are shown in Figure 7.

#### 3.1.2. Accuracy of the UWB Localization

**Figure 8:**
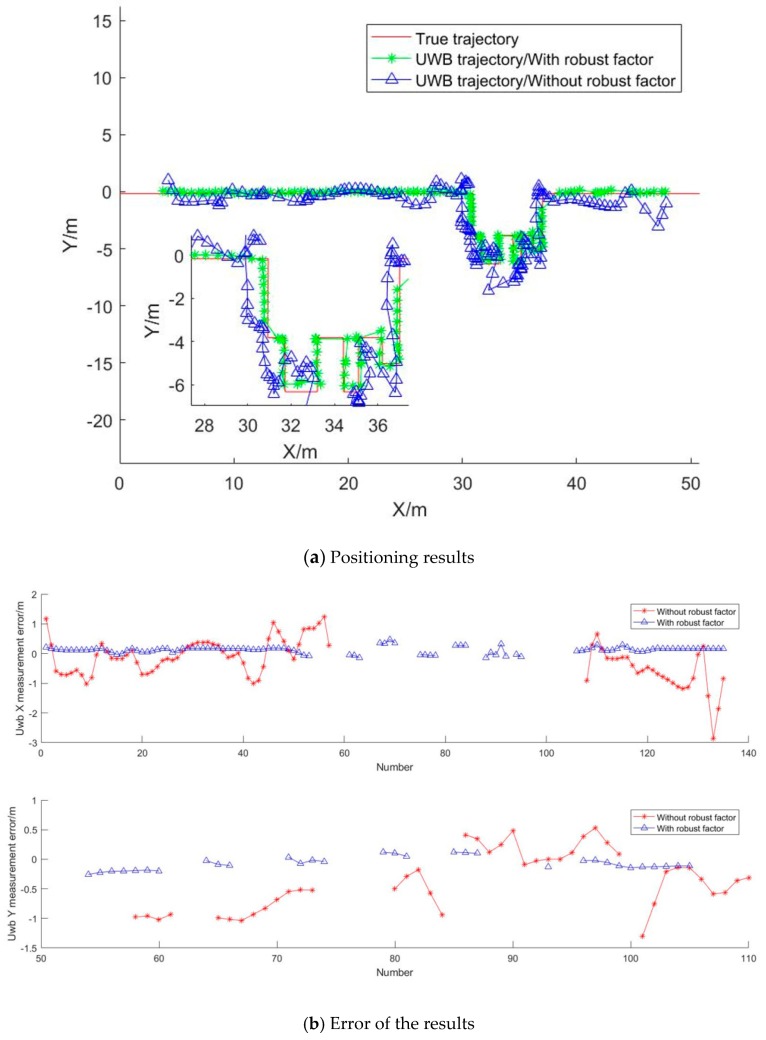
UWB positioning results and errors.

In order to verify the feasibility of the UWB location algorithm proposed, in a scene where the corridor is narrow and long and the UWB anchors are on the same elevation surface, an experimental analysis was made. Two methods for the calculation of the coordinates of the UWB tag are used. One is the least square method, the other introduces the robust factors and ill-conditioned constraint parameters. The results and errors are shown in Figure 8.

From Figure 8, it can be seen that the positioning results solved directly by the least square method are affected by the narrow UWB network and the consistent elevation of the base station; in addition, the positioning results are ill-conditioned and the error is large. Because of the low positioning accuracy, it is difficult to determine the real position, so the accuracy analysis is carried out with the points that can find the corresponding position. In the X direction, the maximum error is −2.86 m, and the RMSE (Root Mean Squared Error) is ±0.61 m. In the Y direction, the maximum error is −1.30m, and the RMSE is ±0.71 m. From the results of local magnification, we can see that it is difficult to judge the real position of the positioning point in the indoor part. However, the positioning results with robust and ill-conditioned constraints have high accuracy and are in good agreement with the real trajectory. In this method, all the positioning points are selected for accuracy analysis. In the X direction, the maximum error is −0.26 m, and the RMSE is ±0.13 m. In the Y direction, the maximum error is 0.48 m, and the RMSE is ±0.16 m. All in all, the positioning accuracy has been greatly improved with the paper’s algorithm.

#### 3.1.3. Results of PDR Localization

**Figure 9:**
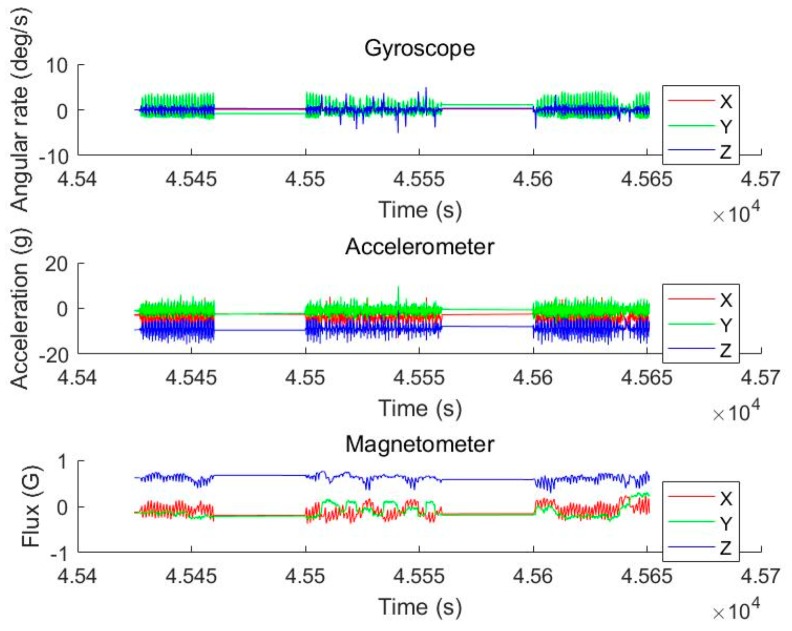
Primitive data obtained by INS devices.

Figure 9 shows the acceleration, angular velocity, and magnetic field intensity data collected by a 9-axis INS device during pedestrian advance in the experiment.

**Figure 10:**
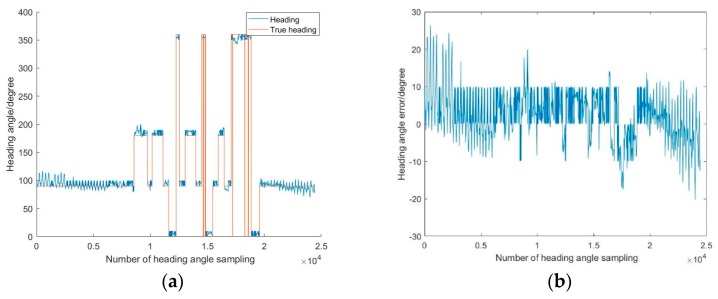
Pedestrian heading and error.

Figure 10 shows the heading angle of the pedestrian calculated by this method (the north direction is the initial reference). As can be seen from the figure, this angle can reflect the direction of the pedestrian. However, in the experiment, the pedestrian walks in a fixed direction and the angle change is small, but the heading angle in the map fluctuates greatly, which indicates that there is a large error; the maximum error is 26.41° and the RMSE is ±6.61°. The dead reckoning method has high requirements for the course angle, so it is necessary to further correct the course angle. For this reason, the heading angle is adjusted by a map constraint. In order to make the positioning error less than the step size, the heading angle cannot exceed 5°. Once the angle is more than 5°, the map heading is used to restrict the heading.

**Figure 11:**
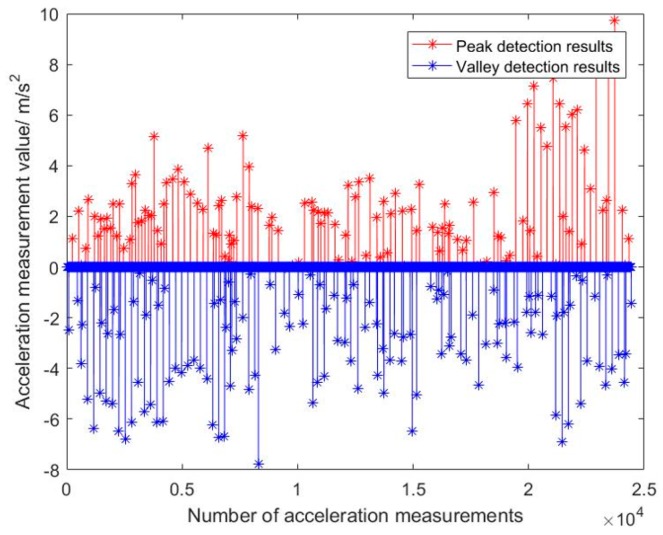
Detection results of Peak and Valley of pedestrian motion acceleration wave.

**Figure 12:**
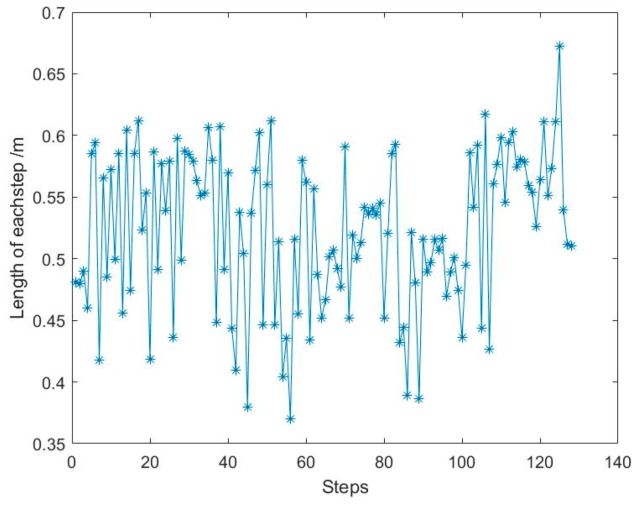
Calculation results of pedestrian motion step size.

Figure 11 reflects the variation of acceleration during pedestrian advance and clearly indicates the maximum and minimum acceleration. Figure 12 is the pedestrian step calculated by this method.

From Figure 11, it can be seen that the acceleration peak and valley of a person can be detected. The maximum acceleration is due to the peak time, and the minimum acceleration is due to the valley time. So the step length can be calculated with Equation (12). The step length is about 0.38~0.68 m and can be seen in Figure 12. The number of steps is 135, which is equal to the true steps. Meanwhile, the total calculated range is about 65 m, which is basically the same distance as the actual walking distance. That is to say, this method can be used to estimate the walking pose of a pedestrian.

#### 3.1.4. UWB/PDR/Floor Map Combined Localization

In order to verify the positioning accuracy of this method, two kinds of experimental scenarios are designed. In the first scenario, the whole area of the UWB base station is used. In the other scenario, the UWB signal is out of lock or unavailable for a certain period of time. In both scenarios, the UWB/PDR/Floor Map data are combined to obtain the positioning information.

##### UWB Full Time Available

**Figure 13:**
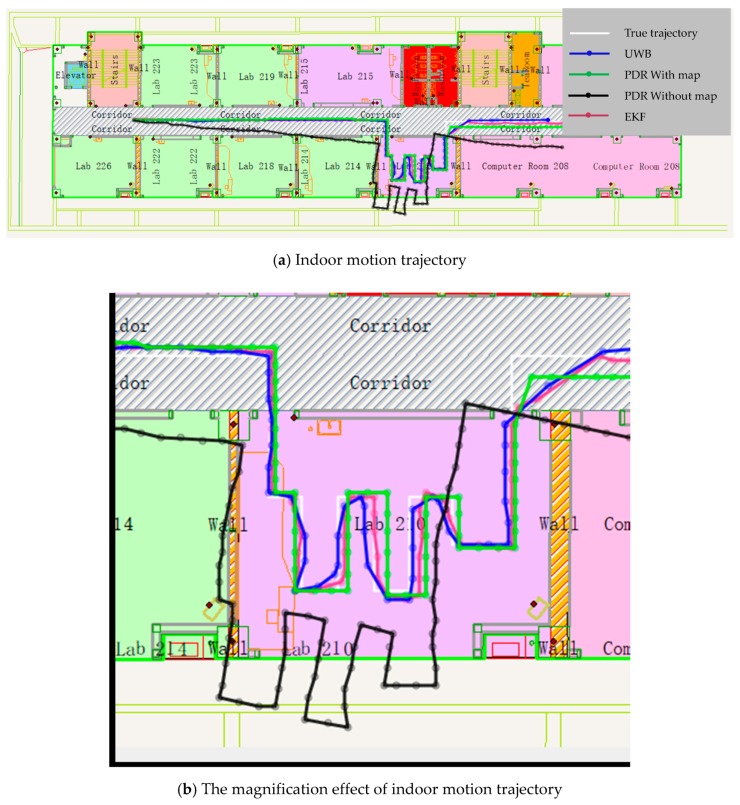
Pedestrian trajectory calculated with four algorithms.

**Figure 14:**
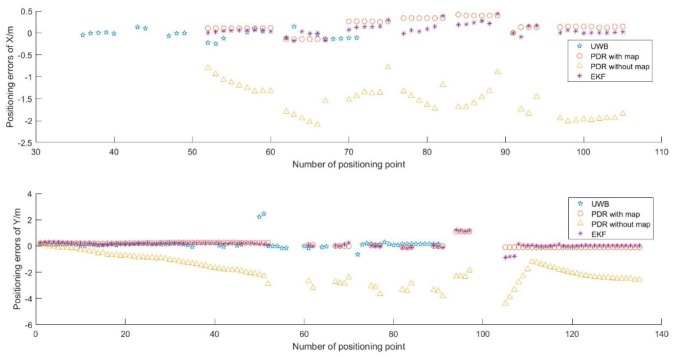
Positioning error of four methods, (**a**) Positioning error in X direction, (**b**) Positioning error in Y direction.

In this experiment, the full coverage UWB positioning network is used, and the UWB positioning data are used to constrain the PDR positioning results. Figure 13 shows the positioning trajectory using UWB, single PDR, PDR with map constrained, and EKF methods. Figure 14 shows the positioning accuracy analysis of the four methods.

From Figure 13 and Figure 14, we can see that different trajectories are calculated with four algorithms. The positioning method of UWB can realize high accuracy results; the RMSE is ±0.12 m in X direction and ±0.22 m in the Y direction. However, the positioning frequency is low; especially at the turning point, there are jumping phenomenon of position points. Meanwhile, the accuracy has a serious relationship with the quantity of anchors. Although the positioning method of single PDR without map information can make a position results, the trajectory is out of range of the room, and the degree of deviation is increasing. The RMSE is about ±1.55 m in X direction and about ±2.06 m in Y direction. The positioning accuracy is too worse to location. The positioning method of PDR with map information can achieve good location results and a higher frequency relative to UWB, but there is absolute displacement related to the true trajectory. The RMSE is about ±0.25 m in X direction and about ±0.40 m in Y direction. The EKF algorithm can combine all the above data and realize a result that is most consistent with the real trajectory. The RMSE is about ±0.15 m in X direction and about ±0.18 m in Y direction. Meanwhile, the frequency is the same as the PDR.

##### UWB Is Not Available

**Figure 15:**
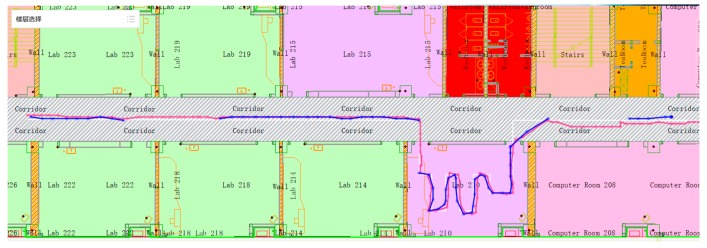
Pedestrian trajectory with UWB unlock 5 seconds*3 times.

**Figure 16:**
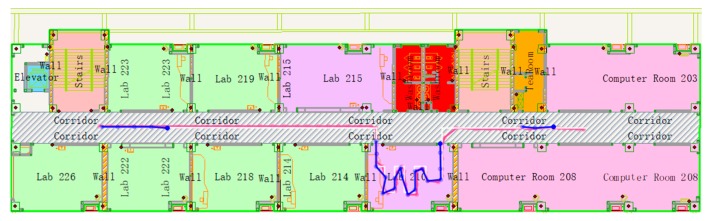
Pedestrian trajectory with UWB unlock in most of the corridor.

Figure 15 shows an experiment that the UWB signal is not available in 3 times, where the blue line disappears, and each time interval is 5 seconds. Figure 16 shows an experiment in which UWB signals were collected only at both ends of the corridor and indoors. There is no UWB signal in most of the corridor.

It can be seen that after the UWB is unlocked, the fusion positioning accuracy is still higher than the map-constrained PDR positioning accuracy within 1 ≤ 2 seconds, and then the accuracy will rapidly decline to the map-constrained PDR positioning accuracy. It can be seen that using the positioning method in this paper, after the pedestrian enters the room, the UWB can be used to initialize quickly to obtain the high-precision initial position of the pedestrian. Based on the map information, the possible walking direction of the pedestrian is obtained, and the heading data on the map is used to constrain the pedestrian’s forward direction. Even without the UWB signal, the positioning accuracy can still be maintained. In Figure 15, the RMSE is about ±0.11 m in X direction and about ±0.20 m in Y direction. In Figure 16, the RMSE is about ±0.38 m in Y direction. In the case of the UWB signal, the fusion positioning is recovered quickly, and the higher positioning accuracy is obtained. This positioning scheme, compared with the pure UWB method, can reduce the number of UWB base stations; compared with the PDR positioning method, high-precision pedestrian motion indoor positioning data can be obtained.

### 3.2. Summary of the Experiment

The novelty of this paper is that a high precision indoor pedestrian location scheme based on UWB, PDR and Floor Map is proposed. First of all, the problems of pedestrian motion initialization and positioning error correction are solved by setting up UWB base stations in narrow corridors and indoors. The positioning accuracy of UWB is great improved from the order of 0.65 m to 0.2 m by adopting Tukey’s robust weight function and ill-conditioned factor into the least square model. Secondly, the PDR method is used to solve the problem of continuous positioning in the environment where the UWB signal is weak or not. The PDR positioning accuracy supported by low-cost INS based on a map assistance strategy is improved from the order of 1.8 m to 0.4 m. Then, the fusion of three kinds of data is used to realize the high precision positioning of pedestrians in a large range of complex indoor environments. Based on the EKF positioning model for multi-source data fusion, when all the UWB is available, the indoor positioning accuracy is further improved to the order of 0.15 m. The UWB signal is unlocked for a long time; the positioning accuracy can still be maintained at the order of 0.38 m. This scheme can not only reduce the number of UWB anchors and reduce the cost and the preparation of UWB anchors deployed, but also ensure high positioning accuracy, and can be suitable for a narrow and long indoor environment with a large area.

## 4. Conclusions

Aiming at the problem of pedestrian location in large buildings, this paper puts forward a data fusion method of UWB/PDR/Floor Map for hybrid pedestrian indoor localization. Mainly, a robust algorithm for UWB positioning is proposed, a heading angle computed strategy for PDR is designed, and an EKF algorithm for fusing UWB/PDR/Floor Map data is suggested. The experiment results indicate that the indoor positioning accuracy can be decimeter-level and the scheme is robust and reliable. This scheme can not only combine the complementary advantages of all three techniques, but can also reduce the quantity of UWB anchors. However, because of the linear movement of people in this paper, the heading angle can be modified by the map data correctly. Therefore, the further research for indoor location will focus on improving positioning results with the introduction of random movement and the in-depth application of map information.
